# Management of Aneurysmal Subarachnoid Haemorrhage and its Complications: A Clinical Guide

**DOI:** 10.4274/TJAR.2023.231205

**Published:** 2023-06-16

**Authors:** Özlem Korkmaz Dilmen, Vincent Bonhomme

**Affiliations:** 1 Department of Anaesthesiology and Reanimation, İstanbul University-Cerrahpaşa, Cerrahpaşa Faculty of Medicine, İstanbul, Turkey; 2 Department of Anaesthesia and Intensive Care Medicine, Liege University Hospital, Liege, Belgium and Anaesthesia and Perioperative Neuroscience Laboratory, GIGA-Consciousness Thematic Unit, GIGA-Research, Liege University, Liege, Belgium

**Keywords:** Brain ischemia, intracranial aneurysm, pulmonary edema, subarachnoid hemorrhage, vasospasm

## Abstract

Aneurysmal subarachnoid hemorrhage (aSAH) is an emergency that needs prompt diagnosis and treatment with endovascular coiling or surgical clipping of the aneurysm to prevent re-bleeding. In addition to neurologic manifestations, aSAH can cause respiratory and cardiovascular complications. The prevention of hypoxemia and hypercarbia, control of intracranial pressure, and restoration of cerebral perfusion pressure should be the primary aims of early management. Secondarily, the most important causes of persistent neurological deficits and physical dependence in aSAH are vasospasm and delayed ischemia following bleeding. During that period, a focus on the detection, prevention, and treatment of vasospasm should be the rule. Transcranial Doppler allows detection and follow-up of vasospasm, especially in severe cases. Nimodipine is the only drug that has proven efficacy for treating vasospasm. Balloon angioplasty is performed in cases of resistance to medical treatment. Along with angioplasty, intra-arterial vasodilators can be administered. New diagnostic and therapeutic advances will hopefully improve outcomes in the near future.

**Main Points**

• The ruptured aneurysms should be repaired as soon as possible to reduce the risk of re-bleeding by surgical clipping or endovascular coiling.

• Providing normoxemia and normocapnia, reduction of intracranial pressure should be the primary aims of early management.

• The monitoring, prevention and treatment of vasospasm and delayed ischemia following bleeding are essential.

• Pressure or oxygen reactivity index monitor provides determining optimal cerebral perfusion pressure (CPP).

• Keeping patient CPP in their optimum autoregulation range may improve long-term neurological outcomes.

## Introduction and Epidemiology

Aneurysmal subarachnoid hemorrhage (aSAH) is mostly caused by the rupture of saccular aneurysms and its global incidence ranges between 2 and 16/100,000 persons a year.^1^ SAH constitutes 2 to 5% of all strokes, and its mortality rate is estimated to range between 32 and 67%. Half of the survivors lives with varying degrees of physical dependence.^2^ The main modifiable risk factors are smoking, high blood pressure, alcohol, and cocaine abuse. Other risk factors include Finnish or Japanese nationality, female sex, Ehlers-Danlos type IV syndrome, autosomal dominant polycystic kidney diseases, neurofibromatosis type I, Marfan syndrome, a family history of aneurysm, and aSAH.^3,4,5,6^

## Clinical Features

Most intracranial aneurysms remain asymptomatic until they rupture. Generally, at the time of rupture, patients suffer from the worst headache of their life. Other signs and symptoms include nausea, vomiting, stiff neck, photophobia, focal neurologic deficits including cranial nerve palsies, deterioration of consciousness, coma, and seizures. Some of them suffer from unusual headaches some days before the rupture due to episodes of minor bleeding.

## Diagnosis

Patients with suggestive clinical presentation, especially when sudden onset neurological deficits and altered state of consciousness are noted, should benefit from a first intention radiological evaluation in the form of cranial computed tomography (CT) without contrast. The characteristic CT manifestations of SAH are hyperdensities in the subarachnoid spaces and cisterns. In case of equivocal CT, lumbar puncture is traditionally proposed as the next diagnostic approach, but nowadays CT or magnetic resonance angiography (CTA or MRA) are preferred. If SAH is detected on CT, digital subtraction angiography (DSA) is usually performed so that three-dimensional images can be reconstructed and the eventual aneurysm localized.

**Figure 1:**
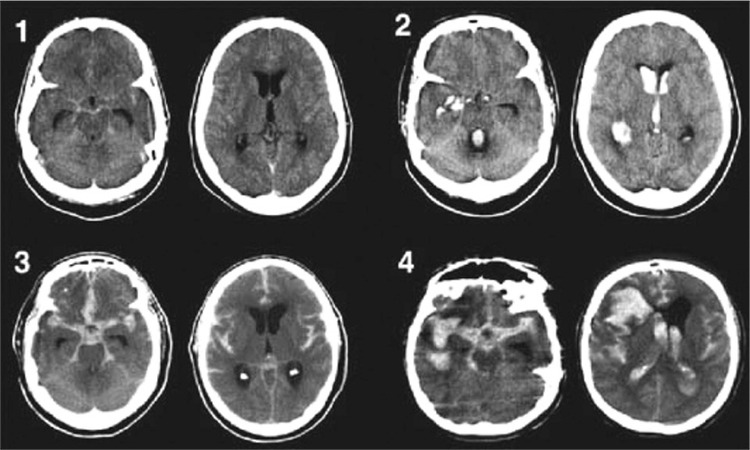
Fisher scale.

The severity of aSAH is categorized using the World Federation of Neurological Surgeons (WFNS) or Hunt and Hess Score grading systems (Table 1, 2).^7,8^ The WFNS score is based on the Glasgow coma score and the presence or absence of motor deficits, while the Hunt and Hess score takes into account associated clinical signs and symptoms. The clinical grade correlates with the severity of the haemorrhage and the risk of subsequent morbidity and mortality. The Fisher grade scale is based on the aspect of blood deposition on CT (Table 3, Figure 1). Higher Fisher grades are associated with a higher incidence of vasospasm and delayed cerebral ischemia (DCI).

## Early Management

Subarachnoid hemorrhage patients have better outcomes in centers treating a large number of them.

Similar to the management of all brain-injured patients, the management of SAH patients should focus on maintaining brain homeostasis and avoid secondary brain injuries of systemic origin. Special attention should be paid to avoid prolonged episodes of anaemia, hypo-or hyperthermia, hypo-or hyperglycaemia, hypo- or hyperoxemia, hypo-hypertension, and hypo-or hypernatremia. Therefore, SAH patients should be monitored adequately and closely in these respects, and adequate measures should be promptly undertaken to correct any detected deviance. Both hypoxemia and hyperoxia have potential harmful effects following brain injury. Lower brain tissue oxygen tension and longer desaturation periods are associated with mortality after SAH.^9^ Hyperoxemia-induced cerebral vasoconstriction, which may accelerate brain tissue ischemia, is well-known fact, but the actual cut off value for oxygen is not clear. Both hypercapnia and hypocapnia are associated with poor neurological outcomes. Hypercapnia elevates intracranial pressure (ICP) by inducing vasodilation and thus potentially impairs cerebral perfusion. Hypocapnia produces vasoconstriction and worsens cerebral vasospasm. However, short-term mild hyperventilation can sometimes be applied to decrease raised ICP, but only transiently. To ensure adequate gas exchange control, patients with a Glasgow coma score less than 9 should be intubated and beneficiated from mechanical ventilation using lung protective ventilation protocols described below in the section on neurogenic pulmonary oedema.

## Management of Raised Intracranial Pressure and Restoration of Cerebral Perfusion

**Figure 2:**
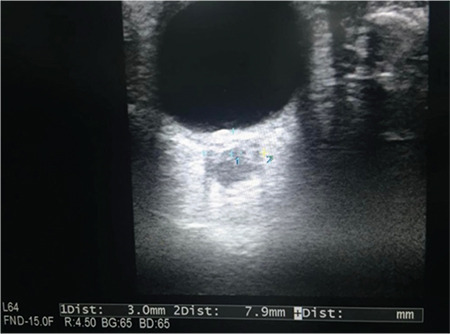
Measurement of optic nerve sheat diameter.

**Figure 3:**
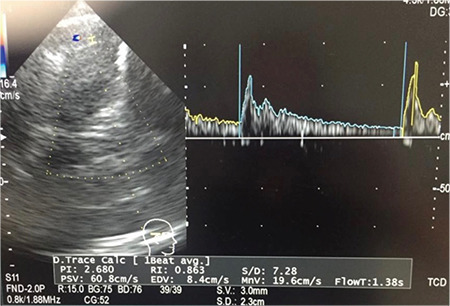
Transcranial Doppler in elevated intracranial pressure.

Acute elevation of ICP following aSAH causes acute neurologic deterioration in patients. Therefore, it is of utmost importance to detect it. Non-invasive optic nerve sheath diameter (ONSD) measurement by ultrasonography and transcranial Doppler (TCD) provide rapid diagnosis of raised ICP. More than 6 mm ONSD indicates elevated ICP (Figure 2). When a decreased diastolic flow velocity and increased pulsatility index above 2.13 are observed with TCD, a raised ICP should be suspected (Figure 3). The pulsatility index is a reflection of peripheral resistance, which is equal to the difference between the peak systolic velocity and end diastolic velocity divided by the mean velocity. A diastolic flow reversal indicates a severely elevated ICP.^10^

The early placement of an external ventricular drain (EVD) and cerebrospinal fluid (CSF) drainage reduces ICP and helps restore CPP. Noteworthy, excessive and rapid CSF drainage can cause an acute increase in the transmural pressure gradient at the level of the aneurysm wall and may favor re-bleeding if the aneurysm is not secured by coiling or clamping.^11^

There is no consensus regarding the duration and modalities of CSF drainage in aSAH. In a randomized, controlled study involving 60 patients and comparing intermittent and continuous drainage, EVD occlusion, bleeding, and infection were found to be more frequent in the continuous drainage group of patients. There was no difference between groups in terms of ICP control, occurrence of delayed ischemia, and quality of functional recovery.^12^ CSF drainage by EVD or lumbar catheter reduces the risk of vasospasm, especially in aSAH cases with high Fisher grades.^13^ Osmotic diuretics, mannitol, or hypertonic saline can be used in combination with CSF drainage to control ICP and CPP, as well as transient mild hyperventilation, as mentioned above.

The optimal CPP is unclear in different phases following SAH. The American Heart Association (AHA) guidelines recommend keeping systolic arterial pressure less than 160 mmHg by using nicardipine, labetalol, or esmolol to prevent re-bleeding until surgical clipping or endovascular coiling are performed.^14^ The aggressive management of blood pressure reduces the risk of re‐bleeding, however, at the expense of an increased risk of secondary ischemia. In general, hypertension is advised following surgical clipping or endovascular coiling to prevent DCI, but the efficacy of induced hypertension has not been shown by randomized controlled studies.^15^ There is an interindividual variability in cerebral autoregulation (CA). The continuous evaluation of CA has been developed by measuring the pressure reactivity index that calculates the Pearson correlation coefficient between the ICP and the mean arterial pressure. The oxygen reactivity index uses brain tissue oxygenation as a surrogate for cerebral blood flow. In addition, the measurement of cerebral oxygen saturation with near-infrared spectroscopy has been used as a continuous autoregulatory index. The cortical spreading depolarization (CSD) occurrence is correlated with impaired CA and may represent the pathological mechanism of DCI in patients with impaired CA. Keeping patients’ cerebral perfusion pressures in their optimum CA range following aSAH may lead to a decreased incidence of CSD and improve long-term neurological outcomes.^16^

The occlusion of CSF passageways by blood or blood products and adhesions produces acute and chronic hydrocephalus. The patient’s age (>65 years), the location of the aneurysm (anterior circulation), the Hunt and Hess IV and V class patients, and the amount of blood in the subarachnoid space and ventricles (Fisher III, IV class) are risk factors for developing shunt-dependent chronic hydrocephalus.^17^

The risk of developing hydrocephalus has been found to be higher after endovascular control of aneurysm compared with surgical clipping. Continuous CSF drainage using a lumbar catheter reduces the risk of hydrocephalus in patients whose aneurysm is excluded through an endovascular procedure.^18,19^

## Prevention of Re-bleeding

Re-bleeding occurs in 4 to 13.6% of cases, and most frequently during the first 24 h after aSAH.^20,21,22^ Early securing of the aneurysm by coiling or clipping is the treatment of choice to prevent re‐bleeding. High grade on admission, maximal aneurysmal diameter, and hypertension increases the risk of re-bleeding. Hypertension should be treated until surgical clipping or endovascular coiling is performed. The aggressive management of blood pressure reduces the risk of re‐bleeding, however, at the expense of an increased risk of secondary ischemia. The AHA guidelines recommend keeping systolic arterial pressure less than 160 mmHg by using nicardipine, labetalol, or esmolol until surgical clipping or endovascular coiling are performed.^14^

In patients chronically using anticoagulants, the treatment should be discontinued. The anticoagulant effects of warfarin or other anti-vitamin K agents should be reversed using prothrombin complex concentrates (50 U kg^-1^) and vitamin K (10 mg IV) or fresh frozen plasma (10-15 mL kg^-1^ IV). Idarucizumab is used to antagonize dabigatran effects. There is no specific antidote for rivaroxaban, apixaban, and edoxaban, but prothrombin complex concentrates (50 U kg^-1^) are recommended to partially antagonize their effects.^23^

The systematic intravenous administration of tranexamic acid (usual dose 10-15 mg kg^-1^ over 20 min) to reduce the risk of re-bleeding following aSAH should be considered. A meta-analysis examining the results of 8 studies investigating the use of tranexamic acid in SAH revealed that re-bleeding decreased in all study groups receiving the agent. One study found an increased risk of ischemia after tranexamic acid administration, while no difference was found in the other study. Tranexamic acid administration decreased mortality at a statistically insignificant level.^24^ Therefore, tranexamic acid or aminocaproic acid administration is recommended, particularly in case of delayed surgery or endovascular intervention to exclude the aneurysm.

## Aneurysm Repair

Ruptured aneurysms should be excluded from the cerebral circulation as soon as possible to reduce the risk of re-bleeding. This can be performed through surgical clipping or endovascular coiling.^14^ Although some studies have shown that endovascular coiling reduces mortality and morbidity compared to surgical clipping, the choice of the aneurysm exclusion technique should not be done blindly. The size of an eventual hematoma and the location, size, and shape of the aneurysm should be taken into consideration.^25,26^ For example, large neck aneurysms can sometimes not be approached through an endovascular technique. In addition, the experience of the team is essential for the decision.

## The Treatment of aSAH-related Complications

### Vasospasm and Delayed Ischemic Deficit

The most important cause of neurological deficit in aSAH is vasospasm in the cerebral arterial circulation and delayed ischemia. Vasospasm most commonly occurs as a result of segmental or diffuse macro and microspasms in the cerebral circulation between days 5 to 15 (may extend up to day 21) following bleeding. While the rate of vasospasm detected by angiography ranges between 70 and 90%, symptomatic vasospasm is seen only in one-third of cases.^27,28^ Higher Fisher grades are associated with higher risk of vasospasm and DCI.

Pathophysiologically speaking, oxyhemoglobin, free oxygen radicals, and neuro-inflammation are held responsible for an increased secretion of endothelin-1, which is a potent vasoconstrictor.^29^

Newly developed neurologic deficits should suggest vasospasm; clinical changes that may be due to other causes such as fever, leucocytosis, and hyponatremia should not preclude searching for a vasospasm, insofar as those events can also be seen during the vasospasm process.

The definitive diagnosis of vasospasm is performed by DSA; however, since TCD is non-invasive, and easily performed at the bedside, daily serial measurements using that technique are recommended to improve the detection of vasospasm before its clinical manifestations and to ensure its follow-up. It also helps determine the need for angioplasty and/or intra-arterial vasodilator application.

In high WFNS and high Fisher class patients, a progressive increase in the mean velocity rate within the middle cerebral artery (MCA) during the early stage of SAH indicates vasospasm. The normal MCA mean velocity rate was less than 80 cm s^-1^. Mild vasospasm velocity rates are considered to range between 120 and 159 cm s^-1^, moderate vasospasm between 160 and 199 cm s^-1^, and severe vasospasm over 200 cm s^-1^. Symptomatic vasospasm is often seen at mean velocities of 160 cm s^-1^.^10^

The Lindegaard ratio corresponds to the ipsilateral MCA mean velocity divided by the ipsilateral extracranial internal carotid artery velocity. A Lindegaard ratio more than 3 indicates vasospasm, between 3 and 5 mild vasospasm, and more than 6 severe vasospasm.^30^

Nimodipine is the only drug that has been proven to be effective for treating vasospasm. Oral or enteral administration (4x60 mg a day) is more effective than intravenous administration. This calcium channel blocker dilates the arteries, reduces calcium-induced excitotoxicity, and decreases platelet aggregation. Nimodipine treatment should be started within 48 h after bleeding and continued for 21 days. If hypotension develops, vasopressor administration should be started without stopping nimodipine administration.^14^

Early targeted fluid therapy guided by preload and cardiac output monitoring in SAH patients with a high WFNS score reduces the risk of vasospasm and provides better functional outcomes.^31^ Because a positive fluid balance adversely affects survival in SAH, the primary aim should be to provide euvolemia.^32^

The optimal hemoglobin (Hb) concentration in patients with SAH is debated. However, a Hb threshold of > 8-10 g dL^-1^ has been advocated in the latest recommendations.^33^

Cerebral microdialysis (CMD) is an invasive neuromonitoring bedside technique using a catheter with a semipermeable membrane probe placed in the brain parenchyma, lactate (L), pyruvate (P), and glucose to enter the perfusate and to be analyzed at hourly intervals. It would be used especially for poor-grade mechanically ventilated SAH patients. CMD can detect energy metabolic changes up to 16 h before DCI, potentially enabling the clinician to provide interventions.^34^ When the LP ratio indicates ischemia, i.e., an increase in the LP ratio in the presence of low P, CPP augmentation is a therapeutic option. When the LP ratio is increased in the presence of low brain tissue oxygen, improving oxygen delivery via increasing the cerebral perfusion pressure, increasing inspired concentration of oxygen, and/or correcting anemia should be considered.^35^

Balloon angioplasty is performed in cases of vasospasm resistant to medical treatment. Along with angioplasty, intraarterial nimodipine, nicardipine, verapamil, and milrinone have also been used in cases of vasospasm. Among the aforementioned drugs, nimodipine is the most promising; however, further studies are needed.^36^

Historically, cerebral vasospasm has been advocated as the underlying mechanism of DCI, but studies targeting vasospasm have failed to reduce its incidence. This has driven a search for other involved mechanisms. The microcirculatory dysfunction in the cerebral parenchyma and coagulation alterations as well as fibrinolytic cascades alterations facilitate the development of microthrombosis that plays a role in the development of DCI after SAH. CSD can be observed and is thought to be related to arteriolar vasoconstriction and inverse neurovascular couplings. The high incidence of CSD following SAH increases the risk of DCI. Another process playing a significant role in the development of DCI is neuro-inflammation, which begins at the moment of arterial rupture and involves microglia. Several mechanisms have been investigated to better understand the pathophysiological pathways and find treatment options for DCI, but the complete picture is not yet clear.^37^

## Seizures

Seizures develop at a rate of 10 to 20% in aSAH. They may further deteriorate the patient’s clinical status. Therefore, anticonvulsant treatment is usually started early after aSAH. However, as the long-term use of anticonvulsants leads to cognitive dysfunction, their prophylactic use raises questions. Currently, it is recommended to initiate prophylactic anticonvulsant therapy in SAH cases where cerebral oedema, or intracerebral or subdural hematoma is evident on CT.^14,36^

## Pulmonary Complications

Aneurysmal SAH may cause acute neurologic pulmonary oedema (NPE). The reported prevalence of NPE after SAH is highly variable in the literature and ranges between 27% and 40%.^38,39^ Several pathophysiological mechanisms underlying the development of NPE have been described. SAH-induced elevated ICP is responsible for reduced CPP. The possible explanation for overactivation of the sympathetic nervous system because of elevated ICP is the stimulation of the hypothalamus and medulla oblongata. Sympathetic discharge increases the circulating catecholamine concentration and produces pulmonary and systemic vasoconstriction. Pulmonary vasoconstriction raises the pulmonary artery pressure, which in turn leads to an increase in pulmonary capillary permeability and ultimately to pulmonary oedema. Elevated ICP also triggers the release of cytokines, which worsens pulmonary capillary leakage.

NPE develops within minutes or hours in most cases, although a delayed presentation can be seen at 12 to 24 h following the central nervous system insult. The clinical manifestation consists of dyspnea, hypoxemia, tachypnea, rales, and excessive foamy secretions in the endotracheal tube. Symptoms generally resolve within 48 to 72 h following the initiation of the elevated ICP treatment and of the supportive treatment including diuretics and mechanical ventilation. Cardiogenic pulmonary oedema and aspiration pneumonia must be considered as differential diagnoses, especially during the acute phase.

As a rule, when mechanical ventilation is needed to prevent hypoxemia and hypercapnia, lung protective ventilation strategies should be used. They consist of protective tidal volumes (6 to 8 mL kg^-1^) along with respiratory rate to avoid hypercapnia and its related increase in cerebral blood flow and ICP. Hypocapnia should also be avoided because of the associated risk of cerebral ischemia. Consequently, normocapnia (PaCO_2_ between 35 and 40 mmHg) should be targeted. Hyperoxia has negative effects on survival after SAH. Hypoxemia (PaO_2_ <60 mmHg and peripheral oxygen saturation <90%) can be prevented by optimizing the inspired oxygen fraction and positive end-expiratory pressure (PEEP). In normovolemic patients, if the patient’s lung compliance is low, the application of PEEP up to 15 cm H_2_O does not increase ICP. In the case of PEEP-induced low blood pressure, CPP may be impacted, and this should be prevented by fluid therapy and vasopressors.^40,41,42^

Fluid therapy can be guided with daily pulmonary ultrasonography evaluation, which can reveal B lines reflecting pulmonary oedema and interstitial fluid in patients with SAH.^43^ Monitoring the cardiac output via transpulmonary thermodilution and pulmonary extravascular lung water (EVLW) can also be a guide for fluid therapy.^44^ In lung oedema, EVLW increases either because of increased lung permeability or because of increased hydrostatic pressure in the pulmonary capillaries, or both. Transpulmonary thermodilution also provides the pulmonary vascular permeability index, which is an indirect reflection of the integrity of the alveolocapillary membrane. Fluid administration should be limited when EVLWI is already high.

## Cardiovascular Complications

Electrocardiographic (ECG) changes such as ST-T wave changes, stress cardiomyopathy, and arrhythmias may develop due to the increase in sympathetic activity, especially in patients with high WFNS scores following aSAH. The incidence of ECG changes is 75% and the incidence of abnormal echocardiographic findings is 17% in patients with SAH. After the aneurysm is excluded, ECG changes often improve on the first following day, and ejection fraction and regional myocardial wall motion abnormalities improve on the second day.^45^ Regional movement disorder in the myocardium and an increase in troponin I and brain natriuretic peptide are observed when stress cardiomyopathy is present (Takotsubo cardiomyopathy, neurogenic stunned myocardium). Supportive therapy with inotropes is the predominant treatment for stress cardiomyopathy.

## Venous Thromboembolism

The incidence of venous thromboembolism is high in patients with aSAH and is an important cause of morbidity and mortality. The incidence of deep vein thrombosis (DVT) and pulmonary embolism is 3.4-24%.^46^ Non-pharmacological prevention methods such as intermittent compression devices or compression stockings are usually applied during the early period, and pharmacological thromboembolism prophylaxis is delayed to prevent re-bleeding and allow surgery when needed. Note that pharmacological prophylaxis with low-molecular-weight heparin is recommended at the earliest 24 h after bleeding.^47^

High D-dimer levels, intraparenchymal hematoma, and motor deficits are important risk factors for DVT. The lower extremity venous Doppler should be performed early in patients with these criteria.

## Neuroendocrine Disorders

The most common metabolic problem in aSAH is hyponatremia (<135 mmol L^-1^) and the most common reason is cerebral salt wasting. The cerebral salt wasting syndrome is characterized by a hypovolemia and an increase in urine output and urinary sodium concentration (>50 mmol L^-1^). This is due to an increase in brain natriuretic peptide secretion. The treatment of cerebral salt wasting syndrome involves sodium and fluid replacement. In addition, fludrocortisone administration provides sodium reabsorption from the distal tubules. Inappropriate antidiuretic hormone secretion syndrome (SIADH) can also be seen in SAH cases in which the anterior cerebral circulation is involved. Hyponatremia is also evident in SIADH; however, urine output is not as high as in cerebral salt wasting. Fluid restriction is the mainstay of SIADH treatment.

Hypothalamo-pituitary ischemia and diabetes insipidus (DI) may develop due to decreased CPP and/or vasospasm in the anterior cerebral artery. DI results in hypernatremia (>145 mmol L^-1^), increased urine output, and hypovolemia. The synthetic ADH analog desmopressin acetate and fluid replacement were used for its treatment. Hypernatremia is an indicator of the poor prognosis in SAH cases.^14^

The negative effects of hypo- and hyperglycemia on neurological recovery are known. It has been shown that blood glucose levels above 140 mg dL^-1^ increase the risk of adverse outcomes and mortality in SAH patients.

Central fever is a common complication of SAH. The causes of central fever are neuronal damage, presence of blood and blood products in the ventricles and subarachnoid space, vasospasm, and systemic inflammatory response. Fever has negative effects on cognitive recovery; therefore, normothermia should be sustained following aSAH.^48^

## The Role of Neuroprotection after SAH

Neuroprotection might be an additional strategy to limit the extent of irreversible damage to neuronal cells after aSAH. Several drugs, potentially those blocking the excitatory cascade leading to secondary neuronal death, have been investigated to improve patient outcomes.^49^ However, to date, none of them has proven to improve patient outcomes. Amantadine, an N-methyl-D-aspartate receptor antagonist, is widely studied in patients with traumatic brain injury. This substance is promising for the reduction of cognitive dysfunction following SAH.^50^ However, further studies are needed to better understand its mechanism of action and better define the therapeutic window where it could be advantageous.

## Conclusion

Despite improvements in the diagnosis and treatment facilities of aSAH, it is still an important reason for mortality and morbidity. Besides neurological manifestations, aSAH can cause respiratory and cardiovascular complications. The prevention of hypoxemia and hypercarbia, reduction of ICP, and the restoration of CPP should be the primary aims of early management. Rebleeding is the most common reason for mortality during the acute period. Therefore, ruptured aneurysms should be repaired as soon as possible to reduce the risk of rebleeding by surgical clipping or endovascular coiling. The most important cause of neurological deficit and physical dependence in aSAH is vasospasm between days 5 and 15 the following bleeding. We should focus on monitoring, prevention, and treatment of vasospasm during that period. Nimodipine is the only drug that has been proven to be effective for treating vasospasm. Balloon angioplasty is performed in cases of vasospasm resistant to medical treatment. Along with angioplasty, intra-arterial vasodilators can be administered. New diagnosis and management advances will hopefully improve outcomes in the near future.

## Figures and Tables

**Table 1:**
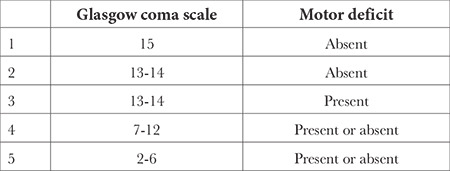
World Federation of Neurological Surgeons Grading Scale

**Table 2:**
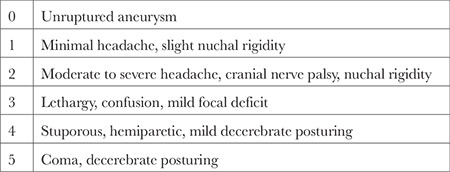
Hunt and Hess Grading Scale

**Table 3:**
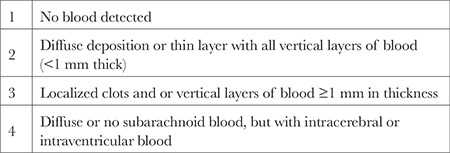
Fisher Scale
